# Early prostaglandin E_1_ treatment improves visual outcomes in central retinal artery occlusion: a retrospective study

**DOI:** 10.3389/fopht.2025.1665519

**Published:** 2025-08-20

**Authors:** Hiroki Sano, Ryoji Yanai, Hirotaka Kondo, Yoshinori Mitamura

**Affiliations:** ^1^ Department of Ophthalmology, Tokushima Red Cross Hospital, Komatsushima, Japan; ^2^ Department of Ophthalmology, Tokushima University Graduate School, Tokushima, Japan

**Keywords:** central retinal artery occlusion, prostaglandin E1, retinal ischemia, optical coherence tomography, visual outcome, neuroprotection

## Abstract

**Background:**

Central retinal artery occlusion (CRAO) is a vision-threatening emergency with no established effective treatment. Prostaglandin E_1_ (PGE_1_), known for its vasodilatory and cytoprotective properties, may offer therapeutic benefits for retinal ischemia.

**Methods:**

In this retrospective study, we compared visual outcomes between CRAO patients who received intravenous PGE_1_ within 24 hours of symptom onset (followed by oral administration) and those who received conventional therapy. PGE_1_ was administered intravenously for 5 days.

**Results:**

At one month, the PGE_1_ group showed significantly better best-corrected visual acuity compared to the control group. Baseline structural retinal parameters, including maximal retinal thickness (MRT) and central retinal thickness (CRT), did not differ significantly between groups. In the PGE_1_ group, baseline MRT was negatively correlated with visual acuity at one month. Retinal arteriovenous diameters showed no significant change post-treatment. No adverse events were observed in either group.

**Conclusion:**

Early administration of PGE_1_ may improve visual outcomes in CRAO. These findings support further investigation into PGE_1_ as a potential treatment for acute retinal ischemia.

## Introduction

Central retinal artery occlusion (CRAO) is caused by obstruction of the central retinal artery, leading to retinal ischemia and sudden vision loss (1, 2). The prognosis for vision recovery is generally poor, often resulting in permanent visual impairment that significantly affects quality of life (1–3). Existing treatments for CRAO, such as ocular message (4, 5), anterior chamber paracentesis (6, 7), intraocular pressure-lowering agents (5, 8), thrombolytic therapy (2, 9), hyperbaric oxygen therapy (10, 11), vitrectomy (12, 13), and neodymium-doped yttrium aluminum garnet laser (14, 15), have shown limited effectiveness in improving visual outcomes, and no established treatment has been confirmed (1, 2, 16).

Prostaglandin E_1_ (PGE_1_) is known for its vasodilatory effects, which improve blood flow in peripheral arterial diseases (17, 18), and has drawn attention as a potential treatment for CRAO (19–21). PGE_1_ acts on the vascular endothelium to increase oxygen supply to the retina, potentially aiding visual recovery (22). It also has neuroprotective properties by reducing oxidative stress and inflammation in ischemic tissue (23, 24).

This study aimed to evaluate the therapeutic effects of PGE_1_ in patients with CRAO. A control group was included to compare outcomes with conventional treatments. We also assessed PGE_1_’s vasodilatory effects by measuring changes in the diameters of the main branches of the central retinal artery and vein before and after administration. Additionally, we analyzed potential prognostic indicators of visual outcomes—including maximal retinal thickness (MRT), central retinal thickness (CRT), and optical-intensity ratio (OIR)—to explore their associations with structural changes and treatment response in the acute phase of CRAO.

## Materials and methods

### Participants

The patient database at Tokushima Red Cross Hospital was searched for individuals diagnosed with acute CRAO between April 2018 and May 2024. Inclusion criteria were cases in which treatment began within 24 h of symptom onset. Exclusion criteria included lack of follow-up data beyond 1 month from onset and suspected arteritic CRAO. From the medical records, we collected data on age at initial visit, sex, time from CRAO onset to PGE_1_ initiation (time to treatment), presence of ocular and systemic diseases, BCVA, and any documented ocular or systemic adverse events. Age, sex, and treatment time were compared between the PGE_1_ and control groups.

### PGE_1_ treatment protocol

Acute CRAO was defined as the rapid onset of vision loss within 24 hours of presentation, with characteristic fundus findings such as ischemic retinal edema and a cherry-red spot in the macula. Subjects in the PGE_1_ group received intravenous infusions of 40 μg PGE_1_ (Alprostadil Alfadex; Takata Pharmaceutical, Saitama, Japan) in 250 mL saline, administered at 125 mL/hour twice daily (80 μg/day) for 5 days, based on prior studies (19). Patients also received 10 μg oral PGE_1_ (Limaprost Alfadex; Sawai Pharmaceutical, Osaka, Japan) three times daily (30 μg/day) for at least 1 month. During the study, no additional treatments (e.g., intraocular pressure-lowering, vasodilating, or thrombolytic agents) were administered. Ophthalmic exams, including slit-lamp biomicroscopy, applanation tonometry, and indirect ophthalmoscopy, were performed daily during the first 5 days and every 1–4 weeks during oral PGE_1_ treatment.

From April 2021 to May 2024, patients who presented within 24 hours of symptom onset were treated with PGE_1_ per this protocol. Before this period, patients received conventional therapy without PGE_1_. Thus, group allocation was based on treatment period rather than physician discretion.

### Outcome measures

The primary endpoint was BCVA comparison at initial visit and at 1 month. Visual acuity was converted to logMAR for statistical analysis. For patients with very poor vision, approximated logMAR values were used: counting fingers = 2.0, hand motion = 2.3, light perception = 2.6, and no light perception = 2.9 (25, 26). Secondary outcomes included: (1) correlations between initial OIR and 1-month BCVA, initial MRT and 1-month BCVA, CRT and 1-month BCVA, and time to treatment and 1-month BCVA; (2) retinal vessel diameters at baseline and 1 month; and (3) adverse events.

### Control group

The control group included CRAO patients who received conventional therapy, including thrombolytics, intraocular pressure-lowering agents, or no PGE_1_. Like the PGE_1_ group, control patients were diagnosed and treated within 24 hours of symptom onset. Inclusion and exclusion criteria were identical to those of the PGE_1_ group. The control group served to assess PGE_1_ efficacy relative to standard treatments.

### Retinal thickness and OIR measurements

Swept-source OCT images were acquired using the Mirante system (Nidek Co., Ltd., Aichi, Japan). MRT, CRT, and OIR were measured only at baseline. MRT was defined as the greatest vertical distance from the inner limiting membrane to the retinal pigment epithelium within a 1.5-mm radius centered on the fovea, manually identified on horizontal B-scans. CRT was the vertical distance at the foveal center. OIR was calculated as the ratio of mean pixel intensity in the inner retina (from the inner limiting membrane to the outer plexiform layer) to that in the outer retina (from the outer nuclear layer to the retinal pigment epithelium), following previously described methods (20), using ImageJ software (National Institutes of Health, Bethesda, MD, USA). A representative illustration of the measurement procedure is provided in **Supplementary Figure S1**.

### Measurement of the retinal vessel diameter

Fundus photographs were used to assess arterial-to-disk (A/D) and venous-to-disk (V/D) diameter ratios. Vessel diameters were measured at the narrowest point of the first superotemporal branch of the central retinal artery between the optic disk and the second branch, and at the corresponding segment of the adjacent central retinal vein. The method was based on previously described techniques (19). Diameters were divided by the vertical optic disk diameter to calculate A/D and V/D ratios, following established methods (20). Measurements were performed at baseline and at 1 month. A representative illustration of this measurement is provided in **Supplementary Figure S2**.

### Measurement protocol and blinding

All parameters (MRT, CRT, OIR, A/D, and V/D) were independently measured by two retinal specialists. One examiner (KH), blinded to visual outcomes, provided measurements used for statistical analysis. The other examiner (HS) was not blinded and contributed data for interrater reproducibility assessment.

### Statistical analysis

Fisher’s exact test was used for categorical variables, such as sex distribution. The Mann–Whitney U test was used to compare continuous variables, including age, time to treatment, BCVA, MRT, CRT, OIR, and A/D and V/D ratios between the PGE_1_ and control groups. To explore prognostic factors for visual acuity in the PGE_1_ group, Pearson correlation coefficients were calculated for BCVA and related parameters. Pre- and post-treatment BCVA changes were evaluated using the Wilcoxon signed-rank test. Paired t-tests were also performed for exploratory analysis, acknowledging the small sample size. Statistical significance was set at p <0.05. All analyses were performed using EZR (Saitama Medical Center, Jichi Medical University; http://www.jichi.ac.jp/saitama-sct/SaitamaHP.files/statmedEN.html).

### Ethics statement

The studies involving human participants were reviewed and approved by the Ethics Committee of the Japanese Red Cross Tokushima Hospital (Approval No. 504). The study was conducted in accordance with local regulations and institutional requirements. The requirement for written informed consent was waived by the ethics committee in accordance with national guidelines and the Declaration of Helsinki, as the study used anonymized retrospective data and obtaining consent was impracticable.

## Results

### Background factors

The baseline characteristics of the PGE_1_ group (n = 4) and the control group (n = 6) are summarized in **Table 1**.

**Table 1:** Baseline characteristics of patients in the PGE_1_ and control groups.

Variable	PGE_1_ Group (n = 4)	Control Group (n = 6)	p-value
Age (years)	73.5 ± 3.87	77.8 ± 7.65	0.33
Male (%)	4 (100%)	3 (50%)	0.2
Time from onset to treatment (hours)	7.5 ± 8.34	7.5 ± 4.84	0.516
Diabetes mellitus (%)	0 (0%)	2 (33.3%)	0.467
Hypertension (%)	2 (50%)	5 (83.3%)	0.5
History of cardiovascular events (%)	1 (25%)	4 (66.7%)	0.524
Atrial fibrillation (%)	0 (0%)	0 (0%)	–
Use of anticoagulant medications (%)	1 (25%)	4 (66.7%)	0.524
COVID-19 vaccination history (%)	0 (0%)	0 (0%)	–

Data are presented as mean ± SD or number (percentage). P-values were calculated using the Mann–Whitney U test for continuous variables and Fisher’s exact test for categorical variables.

“–” indicates that no subject in either group had the corresponding condition; therefore, statistical comparison was not applicable.

No significant differences were observed between the groups in age, sex distribution, time to treatment, or systemic comorbidities, including hypertension, diabetes, and cardiovascular disease. Further granular, individual-level data—detailing baseline and follow-up BCVA, OCT parameters, specific comorbidities, and the treatments administered within the control group are additionally provided in **Supplementary Table S1**.

### Comparison of MRT, CRT, and OIR

**Figure 1:**
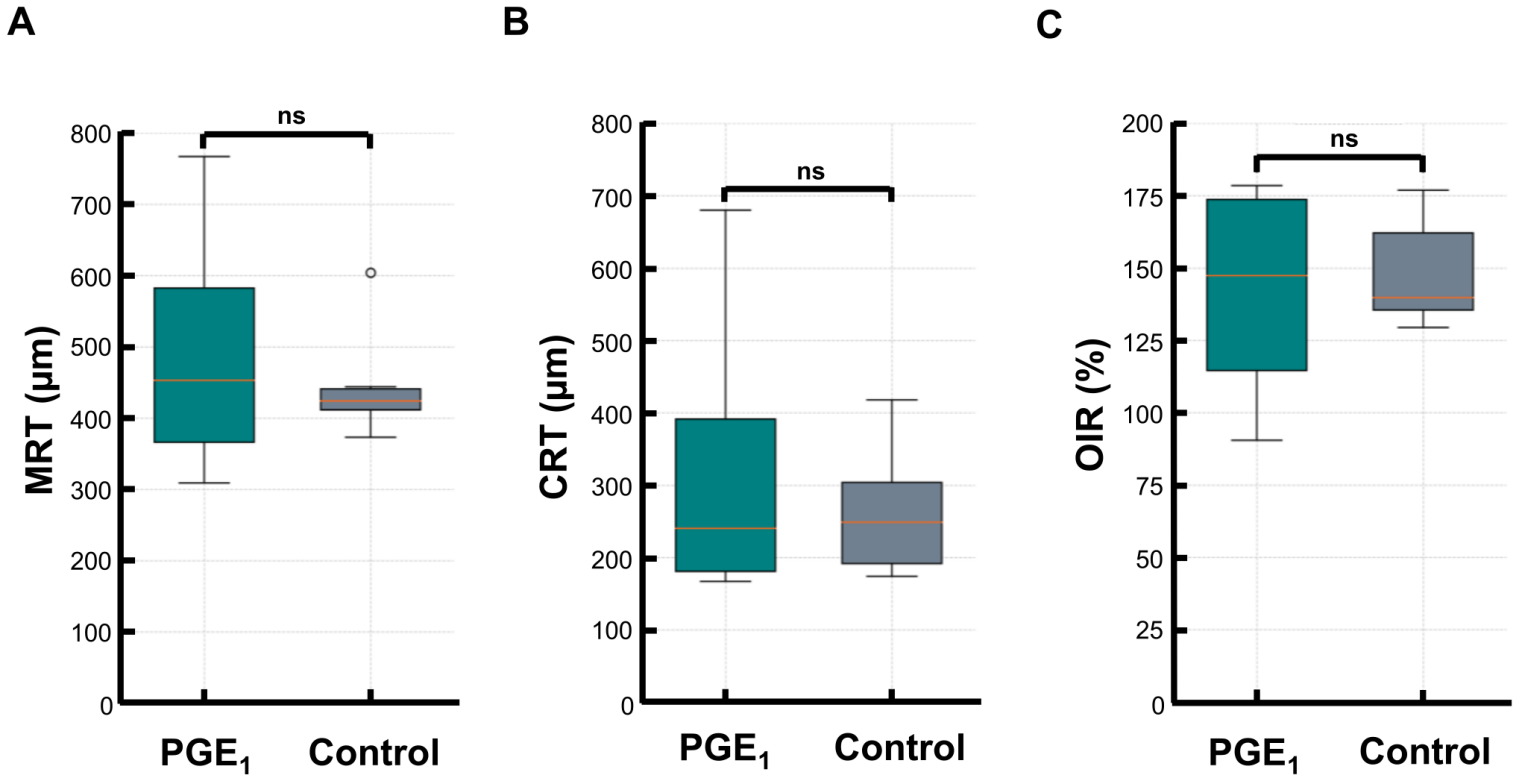
Comparison of MRT, CRT, and OIR at baseline between the PGE_1_ and control groups. Box plots comparing baseline retinal measurements in the PGE_1_ and control groups: **(A)** Maximal retinal thickness (MRT) **(B)** Central retinal thickness (CRT) **(C)** Optical-intensity ratio (OIR) No significant differences were observed between the groups for any parameter.

At the initial visit, the mean MRT was 495.5 (± 174.2) μm in the PGE_1_ group and 446.5 (± 73.8) μm in the control group (p = 1.00). CRT and OIR were also similar: CRT, 331.8 (± 206.9) μm *vs*. 264 (± 85.1) μm (p = 0.91); OIR, 140.9 (± 36.3) *vs*. 148.2 (± 18.0)% (p = 0.91). No significant differences in structural parameters were observed (**Figure 1**).

### Best-corrected visual acuity improvement

**Figure 2:**
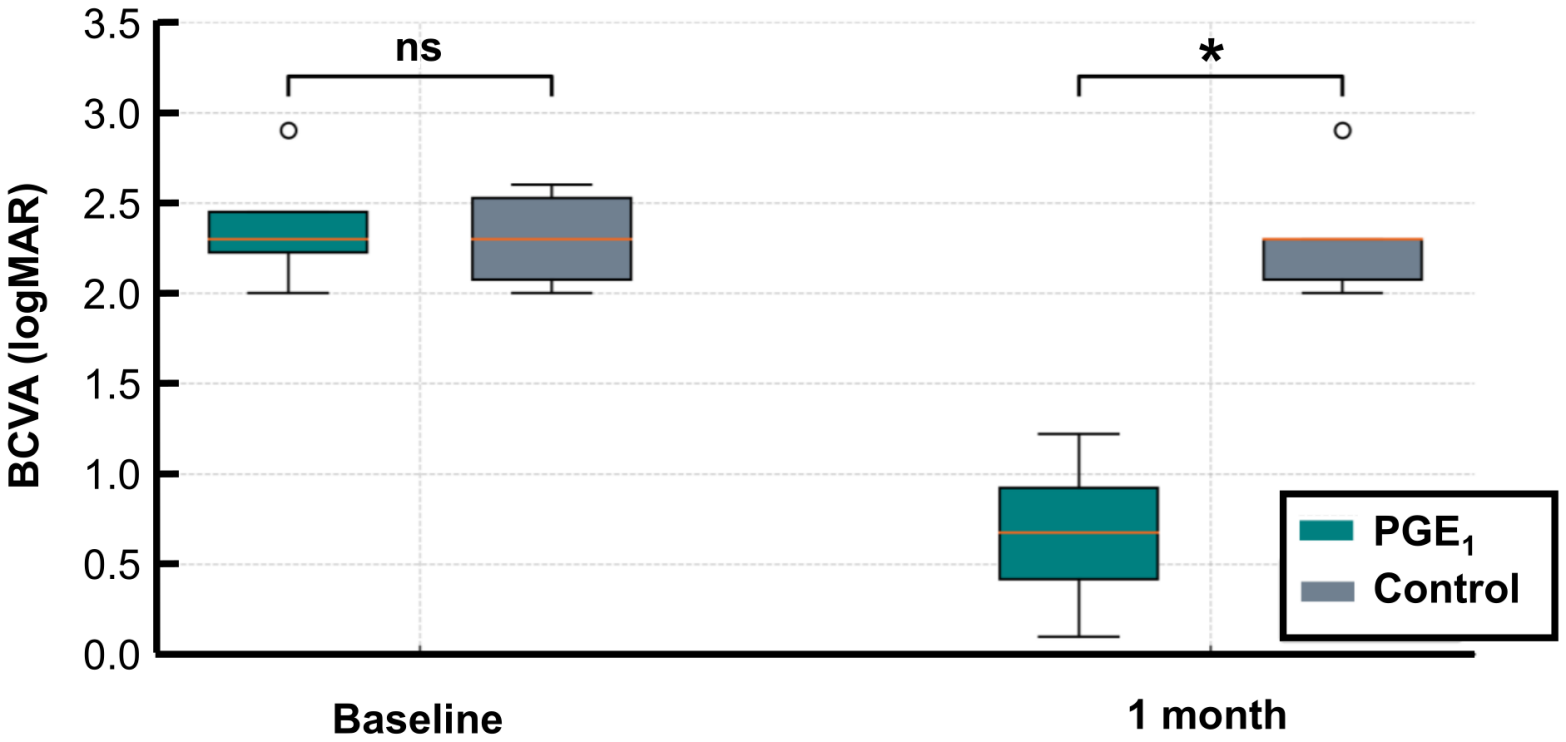
BCVA before and after treatment in the PGE_1_ and control groups. LogMAR BCVA values at baseline and at 1 month are shown for both groups: At 1 month, BCVA was significantly better in the PGE_1_ group than in the control group (*p = 0.013). No significant difference was observed at baseline. *p < 0.05.

At the initial visit, BCVA was 2.4 (± 0.33) logMAR units in the PGE_1_ group and 2.3 (± 0.24) logMAR units in the control group, with no significant difference (p = 0.825). At 1 month, BCVA in the PGE_1_ group was significantly better than in the control group (0.67 ± 0.41 *vs*. 2.3 ± 0.3 logMAR units, p = 0.013) (**Figure 2**).

BCVA improved in all four eyes in the PGE_1_ group. However, the Wilcoxon signed-rank test did not show statistical significance (p = 0.125). The paired t-test showed significant improvement (p = 0.001), but this result should be interpreted cautiously due to the small sample size. No significant BCVA change was noted in the control group using either test.

### Correlation between prognostic factors and visual outcome

In the PGE_1_ group, baseline MRT showed a significant positive correlation with logMAR BCVA at 1 month (r = 0.965, p = 0.034), indicating that a greater retinal thickening in the acute phase was associated with worse visual outcomes.

**Figure 3:**
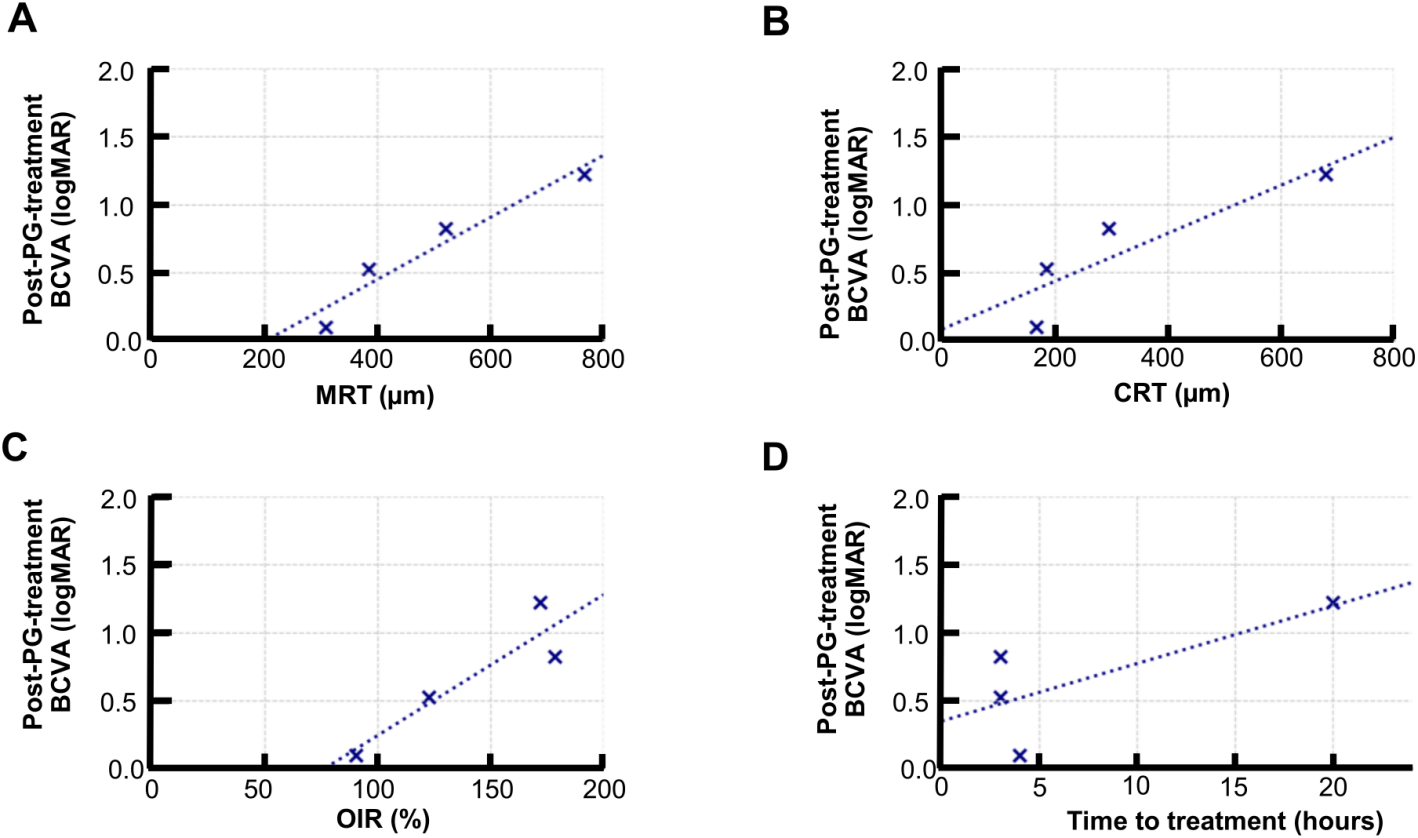
Correlation between baseline retinal structural parameters and visual outcomes in the PGE_1_ group. Scatter plots showing the correlation between baseline parameters and logMAR BCVA at 1 month: **(A)** MRT: strong, significant correlation (r = 0.965, p = 0.035) **(B)** CRT: moderate-to-nonsignificant correlation (r = 0.888, p = 0.112) **(C)** OIR: moderate, nonsignificant correlation (r = 0.914, p = 0.086) **(D)** Time to treatment: moderate-to-nonsignificant correlation (r = 0.745, p = 0.255).

Baseline CRT (r = 0.91, p = 0.08), OIR (r = 0.89, p = 0.11), and treatment time (r = 0.74, p = 0.25) showed moderate positive correlations with BCVA but did not reach statistical significance (**Figure 3**).

Conversely, in the control group, none of the assessed parameters, including MRT (r = 0.14, p = 0.791), CRT (r = −0.0215, p = 0.968), OIR (r = 0.043, p = 0.936), and time to treatment (r = −0.377, p = 0.462), demonstrated a significant correlation with 1-month BCVA, uniformly showing only weak or negligible associations.

### Change in the retinal vessel diameter (A/D ratio, V/D ratio)

**Figure 4:**
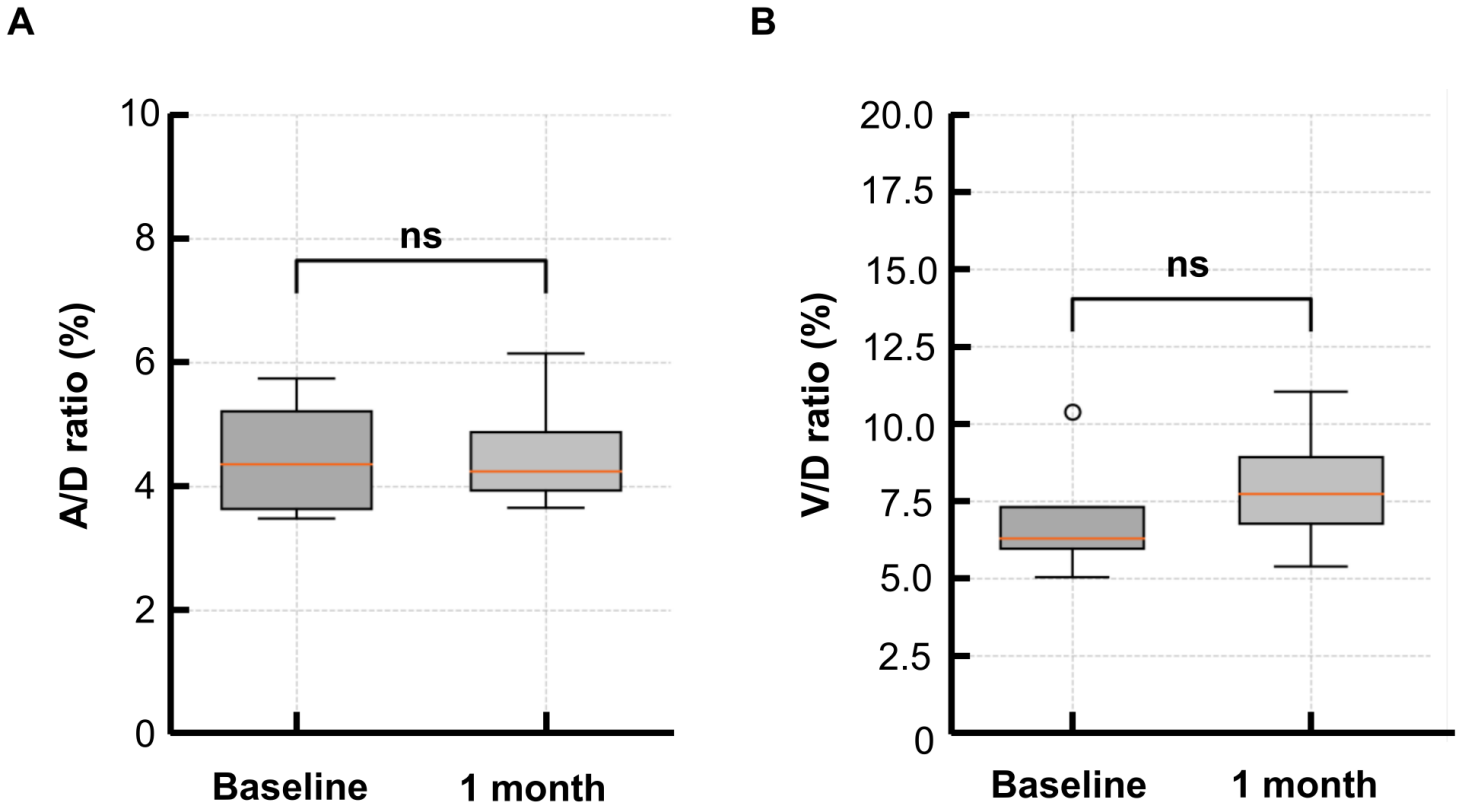
Changes in the A/D and V/D ratios from baseline to 1 month in the PGE_1_ group. **(A)** Arterial-to-disk (A/D) ratio before and after treatment **(B)** Venous-to-disk (V/D) ratio before and after treatment. No significant differences were observed at 1 month for either parameter.

At baseline, the A/D ratio in the PGE_1_ group was 4.5 (± 0.9), and the V/D ratio was 7.0 (± 2.0). At 1 month, the A/D ratio increased to 4.6 (± 1.0), and the V/D ratio to 8.0 (± 2.0). However, no significant changes were observed in A/D or V/D ratios (p = 0.886, p = 0.486) (**Figure 4**).

### Adverse events

Comprehensive monitoring revealed an absence of adverse events or treatment-related complications in the PGE_1_ group, during the administration phase and throughout the follow-up period. Likewise, no serious adverse events were noted in the control group.

## Discussion

This study aimed to evaluate the therapeutic effect of PGE_1_ in patients with CRAO and to identify prognostic factors related to visual recovery. BCVA at 1 month was significantly better in the PGE_1_ group than in the control group. Among structural indicators, baseline MRT showed a statistically significant positive correlation with 1-month BCVA, suggesting that greater retinal thickening in the acute phase was linked to worse visual outcomes. Other factors, such as earlier treatment initiation, lower baseline CRT, and higher OIR, showed moderate correlations with better visual improvement, though not statistically significant.

Several previous studies have reported intravenous PGE_1_ administration for CRAO. Takai et al. administered 40 μg of intravenous PGE_1_ twice daily in 10 patients and observed significant BCVA improvements in all cases (19). Similarly, Malbin et al. reported visual improvement in six patients with acute CRAO after PGE_1_ infusion, with only mild vascular pain and no serious adverse effects (21). Suzuki et al. demonstrated significant visual improvement at 1 and 3 months in 21 patients who received intravenous liposomal PGE_1_ for 7–14 days (20). Chacko et al. documented retinal reperfusion via fluorescein angiography within 48 hours of PGE_1_ infusion in two CRAO cases (27). Our findings support these reports and further strengthen the evidence by including a control group, enabling clearer evaluation of treatment efficacy. Furthermore, it is well-established that intravenous PGE_1_ is generally well tolerated, with commonly recognized side effects including transient flushing, headache, hypotension, and nausea (19–21, 27). Our study likewise observed no adverse events or complications within the PGE_1_ treatment group, thereby further substantiating the favorable safety profile of this intervention in the setting of acute CRAO.

In our study, baseline MRT showed a significant positive correlation with logMAR BCVA at 1 month in the PGE_1_ group, indicating that more severe retinal thickening in the acute phase is associated with poorer visual outcomes. Such a finding positions MRT as a potential structural biomarker reflecting the severity of ischemic injury, particularly relevant in the context of early active therapeutic intervention. Remarkably, this correlation was not observed in the control group, which we attribute to their generally limited visual improvement and the inherent constraints of a small sample size. While MRT offers promising structural insights, comprehensive validation through larger-scale studies is warranted to firmly establish its prognostic utility across a broader spectrum of treatment environments.

The significance of MRT in assessing the severity of CRAO has been underscored in earlier publications. Hayreh et al. emphasized that inner retinal ischemia and subsequent cytotoxic edema are the primary causes of irreversible vision loss in CRAO (28). Supporting this, Ochakovski et al. reported that MRT increased with ischemic edema severity and correlated with visual function, suggesting its value as a functional outcome predictor (29).

Wang et al. showed that visual improvement after intra-arterial thrombolysis was linked to reduced CRT and preserved retinal layer architecture, underscoring the value of OCT structural assessment in CRAO (30). However, Suzuki et al. found no significant correlation between CRT and visual outcomes, highlighting CRT’s limitations as a prognostic indicator (20). In contrast, Kim et al. reported that perifoveal rather than central retinal thickness was more strongly associated with visual recovery, implying that MRT, which reflects the most edematous macular area, may have greater prognostic value than CRT (31). Furthermore, Fouad et al. described that macular fluid in CRAO mainly affects the inner nuclear and outer plexiform layers, reinforcing MRT’s anatomical basis as a marker of ischemic swelling on OCT (32).

Although CRT and OIR showed moderate positive correlations with visual outcomes in our study, neither reached statistical significance. Collectively, these results suggest that MRT may be a more sensitive and clinically relevant structural biomarker for evaluating disease severity and predicting visual prognosis in CRAO than either CRT or OIR.

PGE_1_ is also widely used in other clinical contexts, including treating intermittent claudication in peripheral arterial disease and maintaining ductal patency in congenital heart disease, primarily due to its vasodilatory effects (33, 34). It also protects against ischemia-reperfusion injury in lung transplantation (24). These effects are mediated through E prostanoid receptor activation in vascular endothelial cells, stimulating the cyclic adenosine monophosphate/protein kinase A pathway and relaxing vascular smooth muscle (35). PGE_1_ further activates endothelial nitric oxide synthase and GTP cyclohydrolase I, aiding microcirculation maintenance (36).

However, our study did not detect any significant increase in retinal vessel diameter after PGE_1_ treatment. Recently, PGE_1_ has also shown neuroprotective effects. Zhang et al. reported that PGE_1_ inhibits the c-Jun N-terminal kinase/Bcl-2 interacting mediator of cell death pathway and reduces apoptosis in ischemic tissues (23). Rajan et al. showed PGE_1_ activation of Nurr1, a neuroprotective nuclear receptor (37). Yamamoto et al. found that PGE_1_ upregulates thioredoxin expression via cyclic adenosine monophosphate signaling (38, 39). Additionally, de Perrot et al. demonstrated PGE_1_’s anti-inflammatory effects by suppressing interleukin-1β and tumor necrosis factor-α while increasing interleukin-10 under ischemic stress (24).

Taken together, these pleiotropic effects of PGE_1_ may help explain the better visual outcomes observed in our study, despite the absence of detectable changes in retinal vessel diameter.

This study has several limitations. First, it was a retrospective analysis with a relatively small sample size, which may limit the generalizability of the findings. Second, although OCT-based structural parameters such as MRT were measured by a single examiner (KH) blinded to visual outcomes, manual measurements may still introduce subjectivity. Third, only short-term follow-up data were included, preventing evaluation of long-term effects. Additionally, OCT or fluorescein angiography was not performed to assess retinal perfusion, limiting direct evaluation of vascular changes and reperfusion. Moreover, although BCVA was assessed at one month post-treatment, OCT imaging was not consistently performed at this time point, precluding longitudinal morphological analysis. Previous studies have demonstrated progressive inner retinal thinning following CRAO, which may have prognostic implications (40). Finally, although we evaluated the A/D ratio, we did not assess the ratio of lumen diameter to arterial wall thickness, which might better reflect structural vascular changes. Future studies employing advanced imaging modalities such as adaptive optics scanning laser ophthalmoscopy may help clarify vascular remodeling in response to treatment (41).

Further prospective studies with larger sample sizes, standardized and automated imaging assessments, and longer follow-up are needed to validate these findings and clarify the prognostic value of OCT biomarkers such as MRT.

In conclusion, intravenous PGE_1_ may improve visual outcomes in patients with acute CRAO. Among structural OCT parameters, MRT showed a significant association with visual prognosis and may serve as a useful marker of ischemic severity.

## Data Availability

The original contributions presented in the study are included in the article/**Supplementary Material**. Further inquiries can be directed to the corresponding author.
