# Supplementary material for: Early prostaglandin E1 treatment improves visual outcomes in central retinal artery occlusion: a retrospective study
Source: Front Ophthalmol (Lausanne). 2025 Aug 20;5:1665519. doi: 10.3389/fopht.2025.1665519 (PMC12404978; doi:10.3389/fopht.2025.1665519)
Supplement: Supplementary file 1 [file DataSheet1.pdf]

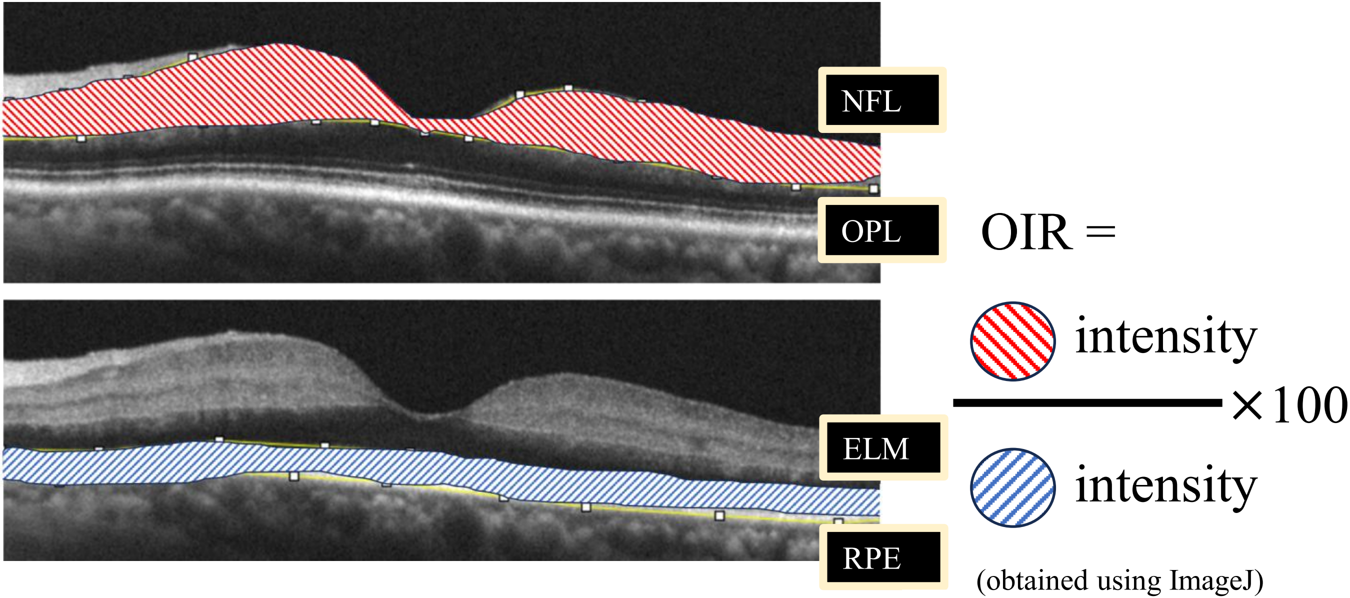

Supplementary Figure S1 Method for OIR measurement. Pixel intensities in the inner and outer retina were quantified using ImageJ, as outlined in the Materials and Methods section.

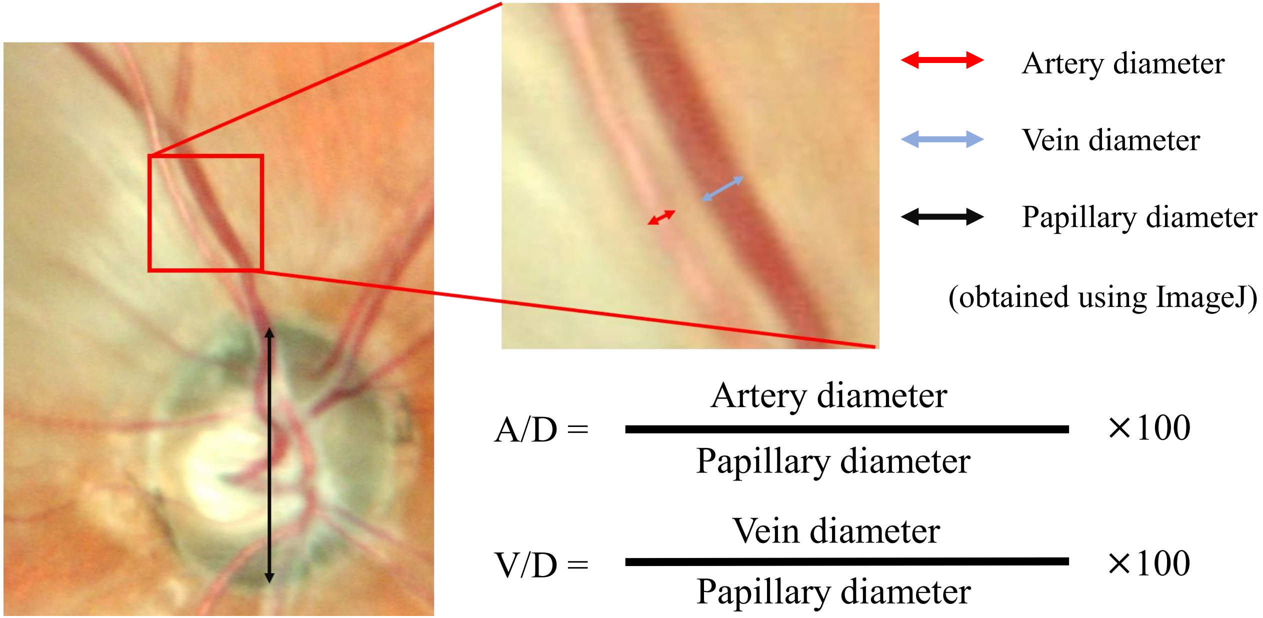

$$A/D = \frac{\text{Artery diameter}}{\text{Papillary diameter}} \times 100$$

$$V/D = \frac{\text{Vein diameter}}{\text{Papillary diameter}} \times 100$$

Supplementary Figure S2 Method for calculating A/D and V/D ratios from vessel diameter measurements. Measurements were conducted using ImageJ, as outlined in the Materials and Methods section.
